# BDNF rs6265 Polymorphism and Its Methylation in Patients with Stroke Undergoing Rehabilitation

**DOI:** 10.3390/ijms21228438

**Published:** 2020-11-10

**Authors:** Massimo Santoro, Mariacristina Siotto, Marco Germanotta, Elisa Bray, Alessia Mastrorosa, Camilla Galli, Dionysia Papadopoulou, Irene Aprile

**Affiliations:** IRCCS Fondazione Don Carlo Gnocchi ONLUS, 50143 Florence, Italy; masantoro@dongnocchi.it (M.S.); mgermanotta@dongnocchi.it (M.G.); ebray@dongnocchi.it (E.B.); amastrorosa@dongnocchi.it (A.M.); cgalli@dongnocchi.it (C.G.); dpapadopoulou@dongnocchi.it (D.P.); iaprile@dongnocchi.it (I.A.)

**Keywords:** BDNF, *BDNF* rs6265 polymorphism, *BDNF* rs6265 methylation, stroke, rehabilitation

## Abstract

Brain-Derived Neurotrophic Factor (BDNF) and its rs6265 single nucleotide polymorphism (SNP) play an important role in post-stroke recovery. We investigated the correlation between *BDNF* rs6265 SNP and recovery outcome, measured by the modified Barthel index, in 49 patients with stroke hospitalized in our rehabilitation center at baseline (T0) and after 30 sessions of rehabilitation treatment (T1); moreover, we analyzed the methylation level of the CpG site created or abolished into *BDNF* rs6265 SNP. In total, 11 patients (22.4%) were heterozygous GA, and 32 (65.3%) and 6 (12.2%) patients were homozygous GG and AA, respectively. The univariate analysis showed a significant relationship between the *BDNF* rs6265 SNP and the modified Barthel index cut-off (χ^2^(1, N = 48) = 3.86, *p* = 0.049), considering patients divided for carrying (A+) or not carrying (A−) the A allele. A higher percentage of A− patients obtained a favorable outcome, as showed by the logistic regression model corrected by age and time since the stroke onset, compared with the A+ patients (OR: 5.59). At baseline (T0), the percentage of *BDNF* methylation was significantly different between GG (44.6 ± 1.1%), GA (39.5 ± 2.8%) and AA (28.5 ± 1.7%) alleles (*p* < 0.001). After rehabilitation (T1), only patients A− showed a significant increase in methylation percentages (mean change = 1.3, CI: 0.4–2.2, *p* = 0.007). This preliminary study deserves more investigation to confirm if *BDNF* rs6265 SNP and its methylation could be used as a biological marker of recovery in patients with stroke undergoing rehabilitation treatment.

## 1. Introduction

Stroke is the primary cause of disability [1] and the second largest cause of death worldwide, with a high burden on patients, their families, and health-care systems [2]. Patients after stroke have a very heterogeneous clinical spectrum, with variable and often incomplete recovery of motor function in response to rehabilitation treatment [3]. Indeed, 30 to 60% of patients present functional deficits of the paretic arm after a rehabilitation program, resulting in impaired activities of daily living [4].

Therefore, the identification of prognostic factors of post-stroke recovery has become an important research field, with the aim of providing a tailored rehabilitation intervention and, consequently, maximizing the amount of recovery. The most commonly used predictors for post-stroke rehabilitation are measures of lesion size [5,6], location of the lesion [7,8], corticospinal tract integrity [9,10], and severity of the initial impairment [11,12]. Indeed, a more severe baseline damage is generally associated with a worse post-stroke recovery [11,12,13], but these factors may explain only a limited amount of post-stroke motor outcome changes and patients’ responses to rehabilitation therapies [14], so it is very crucial to identify novel and more accurate biological markers of recovery.

Recently, different studies have shown the important role of genetic and epigenetic factors in the individual’s capacity for neuroplasticity, and the responsiveness to post-stroke rehabilitation [15,16,17]. After a stroke, the brain has the ability to reorganize its neural networks, through neuroplasticity processes that include both the break-down and rewiring of the existing links, and the creation of new links [2]. In this process, the Brain-Derived Neurotrophic Factor (BDNF), neurotrophin, which shows a very high expression in the brain [18,19,20], seems to have an important role, mainly in neuronal differentiation, survival, and synaptic plasticity. As reported in the literature, the activation of the BDNF signaling pathway is associated with an improvement in motor performance [21]. Thus, the activity-dependent release of BDNF appears to be essential for the motor recovery [22]. BDNF modulation has also been suggested to mediate the therapeutic effect of treatments, increasing perilesional cortex excitability [23,24]. Due to its role in neuronal differentiation, survival, and synaptic plasticity, BDNF has been extensively studied in stroke recovery [25,26,27].

*BDNF* rs6265 is a single nucleotide polymorphism (SNP) G196A that produce a valine (Val) to methionine (Met) substitution at codon 66 (Val66Met) [28]. This SNP modulates intracellular trafficking and *BDNF* secretion, associated with a reduced capacity for use-dependent plasticity in the motor cortex and impaired motor learning [29,30,31]. Moreover, homozygous Met/Met mice showed a greater impairment of post-stroke locomotor functions and reduced angiogenesis [32], even if the association of *BDNF* rs6265 polymorphism with stroke outcomes and recovery remained inconclusive. Some studies on subacute patients after spontaneous recovery have shown a significant association between *BDNF* rs6265 SNP and stroke outcome [27,33]. Regarding the effect of this polymorphism on long-term stroke recovery or response to targeted therapies, Shiner et al. found that, after rehabilitation therapy, the motor improvement of Met carriers (at least one A allele) was significantly less when compared to non-carriers (GG allele) in patients with high or moderate motor function at baseline, while no significant effect of *BDNF* rs6265 on stroke patients with low motor function was found [26].

*BNDF* expression is also regulated by epigenetic mechanisms, such as the methylation of cytosine-guanine (CpG) dinucleotides, with a possible impact on stroke recovery. Indeed, Martinowich et al. found that the hypermethylation of the *BDNF* promoter region may influence stroke outcomes with a decrease in the *BDNF* synthesis [34]. Moreover, Kim et al. showed that higher methylation levels of the *BDNF* promoter region in patients post-stroke were independently associated with incident post-stroke depression (PSD), and were significantly associated with the worsening of depressive symptoms over one year, although there was no correlation with baseline depressive symptom severity [33]. The authors did not find a correlation between *BDNF* rs6265 SNP and methylation levels [33]. These data appear to be discordant with a previous study, which reported that the *BDNF* methylation levels were dependent on genotype with a consequently differential effect on major psychosis [35].

In summary, how *BDNF* genotype and methylation can affect motor function and post-stroke rehabilitation remains uncertain. Here, we investigated whether *BDNF* rs6265 polymorphism and the methylation status of the CpG site into this SNP are involved in post-stroke recovery after rehabilitation treatment.

## 2. Results

The demographic and clinical characteristics of the enrolled sample are reported in Table 1. Forty-nine patients were enrolled and evaluated at baseline. Of those, one patient was not evaluated at the follow-up, because of clinical complications unrelated to the study. Therefore, 48 patients were evaluated after the rehabilitation treatment.

### 2.1. BDNF rs6265genotyping and Methylation Analysis

**Figure 1:**
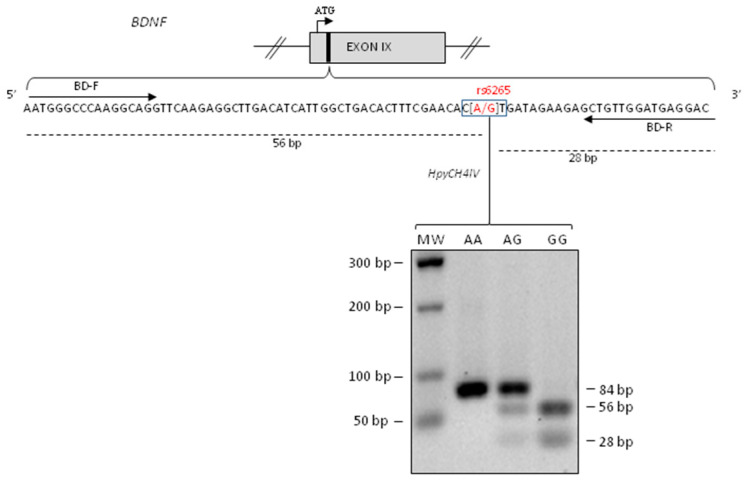
Brain Derived Neurotrophic Factor (*BDNF)* rs6265 polymorphism genotyping. Upper panel: schematic representation of human *BDNF* exon IX (the roman numerals indicate the exon number). The translational (ATG) start site is indicated as an arrow and the region containing rs6265 SNP as a black box. Lower panel: the *BDNF* sequence amplified by PCR using BD-F and BD-R are indicated as arrows [36] while the restriction fragments of HpyCH4IV restriction enzyme as dashed lines. Lower panel: Electrophoresis on 2% agarose gels of PCR fragments after HpyCH4IV digestion. One PCR product of 84 bp indicated homozygous A/A allele, three PCR fragments of 84 bp, 56 bp and 28 bp indicated heterozygous A/G and two PCR products of 56 bp and 28 bp indicated homozygous G/G allele. The size of molecular the weight (MW) markers of the DNA ladder are in the left column.

We performed genotyping for *BDNF* rs6265 polymorphism using PCR amplification followed by the enzymatic digestion with HpyCH4IV in order to identify homozygous GG (fragments of 56 bp and 28 bp), heterozygous AG (fragments of 84 bp, 56 bp and 28 bp) and homozygous AA (fragment of 84 bp) (Figure 1).

We identified 32 (65.3%) patients carrying homozygous GG genotype, 11 (22.4%) patients with heterozygous GA genotype, and 6 (12.2%) patients with homozygous AA genotype.

**Figure 2:**
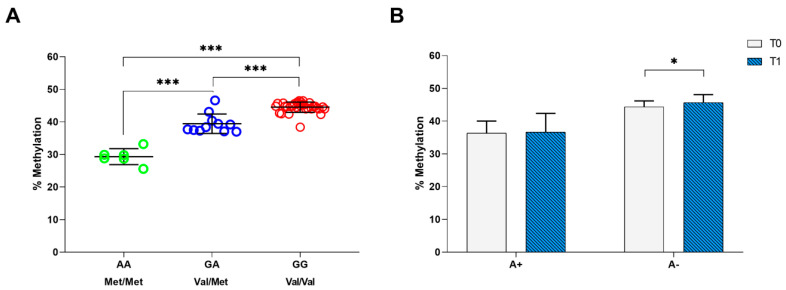
Methylation analysis of *BDNF* rs6265 polymorphism. (**A**) Average percentage of methylation in patients with stroke carrying the GG (Val/Val), GA (Val/Met) and AA (Met/Met) alleles. (**B**) Average percentage of methylation before and after the 30-session rehabilitation intervention for patients carrying A+ and A− alleles. The asterisks indicate a statistically significant difference: *** *p* < 0.001 and * *p* < 0.05, according to the paired *t*-tests.

Moreover, we performed the methylation analysis on the CpG site that can be created or abolished by rs6265 polymorphism. By qRT-PCR on 49 patients with stroke at T0 time point, we found a mean methylation percentage of 44.6 ± 1.1 in patients carrying the GG genotype (Val/Val), 39.5 ± 2.8 in patients with the GA (Val/Met) genotype and 28.5 ± 1.7 in patients with the AA genotype (Met/Met) (Figure 2A). The one-way ANOVA test showed that the levels of methylation were significantly different between *BDNF* rs6265 polymorphisms (F = 142.896, gl = 2, *p* < 0.001), indicating an association between *BDNF* rs6265 polymorphism and methylation; post-hoc analysis highlighted that this difference lied between GG and GA, GG and AA, and GA and AA genotypes, with *p* < 0.001 (Figure 2A).

Finally, we compared the mean methylation percentages of the *BDNF* rs6265 polymorphism (A+ vs. A− alleles) in patients with stroke evaluated at T0 and T1 (*n* = 36) (Figure 2B). We found a significant increase in the mean methylation percentages (~2%) in patients carrying A− (mean change = 1.3, CI: 0.4–2.2, *p* = 0.007), which did not change in patients carrying A+ (mean change = 0.3, CI: −1.4–2.1, *p* = 0.692) (Figure 2B).

### 2.2. Relationship between BDNF rs6265 SNP Analysis/Methylation and Rehabilitation Outcome

**Figure 3:**
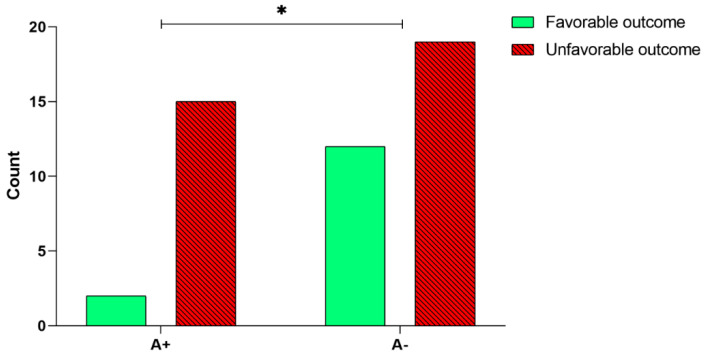
Analysis of *BDNF* rs6265 polymorphism and the rehabilitation outcome. Number of patients carrying A+ and A− alleles that obtained a favorable outcome after the 30-session rehabilitation intervention. The asterisk indicates a statistically significant difference: * *p* < 0.05, according to the χ^2^ test.

In order to analyze a possible correlation between *BDNF* rs6265 polymorphism and the rehabilitation outcome, we first performed a univariate analysis. Our results showed a significant relationship between the A+ or A− alleles and the outcome of the rehabilitation intervention (χ2(1, *n* = 48) = 3.86, *p* = 0.049, Figure 3)—a higher percentage of patients carrying the A− allele obtained a favorable outcome (12 out of 31, 38.7%; Figure 3), compared to patients carrying the A+ allele (2 out of 17, 11.8%; Figure 3). The multivariate analysis by means of the logistic regression model, corrected by age and time since the stroke onset, confirmed that the *BDNF* rs6265 polymorphism (A+, or A− alleles) significantly predicts the outcome (Table 2)—the odds of obtaining a favorable outcome for a patient carrying the A− allele was 5.59 times higher than for patients carrying the A+ allele.

Finally, we report in Table 3 the comparison of methylation percentages at baseline and after the treatment, as well as their changes, between patients with or without a favorable outcome.

No significant differences were detected, except for a trend in T1 evaluation (Table 3).

## 3. Discussion

In recent years, the identification of prognostic factors of post-stroke recovery has become an important research focus aim to develop a more tailored patients’ rehabilitation program. Indeed, some studies have addressed biomarkers involved in the post-stroke recovery [5,8,14].

Epigenetics and genetic variations can play an important biological role in the neuroplasticity process, as well as in modulating the response to a rehabilitation treatment [15,16,17]. *BDNF* rs6265 SNP represents one of the most studied genes for its role in the regulation of neuronal differentiation and plasticity [25,26,27]. The role of rs6265 *BDNF* polymorphism has been extensively studied in animal models, such as knock-in mice homozygous *BDNF* Met/Met, where the up-regulation of angiostatic CD36 and TSP-1 induced a reduction in endothelial cell proliferation, suggesting an association of the Met allele with stroke-induced angiogenic deficits [32]. Moreover, in rats in which an ischemic stroke was induced, the brain BDNF increased [37], and the BDNF up-regulation was associated with motor function improvement [38]. However, it is very difficult to translate data from animal models into human research, with the risk of obtaining controversial results. Although in recent years there has been an increase in clinical and preclinical studies on the role of *BDNF* rs6265 polymorphism in stroke, the effects on the recovery outcome post-stroke remain inconclusive.

In our study, a higher percentage of patients carrying the *BDNF* rs6265 A− allele obtained a favorable outcome, compared with patients carrying the A+ allele (χ^2^(1, N = 48) = 3.86, *p* = 0.049) (Figure 3). The correction by age and time since the stroke onset confirmed that *BDNF* rs6265 polymorphism significantly predicts outcome, with the probability being 5.59 times higher of obtaining a favorable outcome in patients carrying the A− allele compared to those carrying the A+ allele. Note that disability at the baseline was not significantly different between patients carrying A− and those carrying A+. This result means that the two groups of patients (carrying A− and carrying A+), even if they had a comparable disability at baseline, showed a significantly different recovery after rehabilitation. The different responses to rehabilitation treatment of our patients with greater motor independence in those carrying the G allele could be explained with the involvement of the A allele in the neuroplasticity processes. In fact, according to the literature, the A allele is associated with the BDNF dendritic trafficking deregulation, secretory vesicles and synaptic plasticity alteration [29,39].

On the other hand, DNA methylation is an epigenetic mechanism that implies heritable changes of gene expression without a change in the primary DNA sequence. The methylation of CpG sites usually acts to turn off (silence) transcription by recruiting histone deacetylases that induce the formation of inactive chromatin, repressing gene expression [40]. Our results showed that the methylation level of the CpG site included in the *BDNF* rs6265 polymorphism was significantly different between the GG, GA and AA alleles (*p* < 0.001), indicating an association between genotypes and methylation in patients with stroke (Figure 2A). Moreover, we found a significant increase in the methylation levels of *BDNF* rs6265 SNP in patients with stroke carrying the A− allele after rehabilitation treatment (Figure 2B). However, comparing the methylation percentages between patients with or without a favorable outcome, we found no differences at baseline, with a trend, even if not yet significant, after the rehabilitation treatment. This result should be confirmed and possibly reinforced with a greater number of subjects undergoing rehabilitation treatment.

*BDNF* rs6265 methylation has been reported to affect the interaction of transcriptional factors and the relationship between DNA methylation and serum BDNF levels [41]. In this way, the methylation changes associated with *BDNF* rs6265 polymorphism can be differentially linked in both genotypes and BDNF levels [41]. In post-stroke recovery, Kim et al. reported that the high methylation levels of *BDNF* promoter regions were independently associated with long-term but not with acute outcome, and were significantly associated with the worsening of physical disability and cognitive function, measured by BI and the Mini-Mental State Examination (MMSE), respectively [27].

Moreover, the same authors in a second paper showed that the high methylation levels of *BDNF* promoter region were independently associated with post-stroke depression and with a worsening of depressive symptoms during follow-up [33]. Differently from the studies of Kim and colleagues, our study, for the first time, showed the association of *BDNF* rs6265 polymorphism and its methylation with recovery after rehabilitation treatment in patients post-stroke. In particular, from these preliminary data, it appeared that, after a rehabilitation program, A− patients had a more favorable outcome, showing also an increase in methylation levels of *BDNF* rs6265 polymorphism.

The most important limitation of our study is the small sample size, and, therefore, further studies are needed in a larger number of patients to validate our data. Moreover, a larger sample size will allow us to delve deeper into the topic, elucidating the interaction of the *BDNF* rs6265 genotype and methylation with the type of stroke (ischemic or hemorrhagic), or the stroke severity. Finally, we investigated a population with a mean latency since stroke of 90 days, and, therefore, further study should be addressed to investigate the role of the *BDNF* rs6265 genotype in earlier phases of stroke recovery.

Nevertheless, if our preliminary results are confirmed, the *BDNF* rs6265 genotype and its methylation level analysis could become reliable prognostic genetic factors that might help us to better address the optimal treatment for a patient, or to supplement rehabilitation therapy with customized rehabilitation protocol. A better understanding of *BDNF* molecular modulation and its epigenetic regulatory mechanism could impact the management of post-stroke patients, facilitating the selection of a personalized rehabilitation protocol with a greater impact on recovery times, and likely on the reduction of the cost.

## 4. Materials and Methods

### 4.1. Sample

In total, 49 patients with first stroke (28 men and 21 women with a mean age of 68.6 ± 15 years), admitted to our rehabilitation department between 2019 and 2020, were consecutively enrolled.

The inclusion criteria were as follows: (i) first ischemic or hemorrhagic stroke, documented by magnetic resonance imaging (MRI) or computed tomography (CT); (ii) age between 55 and 85 years; (iii) patients able to perform a rehabilitation treatment, for at least 45 min/day, for 5 days/week; (iv) time latency (within 6 months from stroke); (v) cognitive and language abilities sufficient to understand the experiments and follow instructions.

The exclusion criteria were as follows: (i) a previous stroke; (ii) behavioral and cognitive disorders and/or reduced compliance interfering with active therapy.

The study design was approved by the Ethical Committee of Don Carlo Gnocchi Foundation, Milan, Italy on March 13, 2019 (FDG_6_13/3/19). Written informed consent was obtained from all patients after a detailed explanation of the study’s aims and rehabilitation protocols (clinical trials identifier: NCT04223180).

### 4.2. Rehabilitation Treatment and Outcome

Patients underwent a rehabilitation program including conventional physical therapy treatment, performed 6 days a week, lasting 45 min, focused on lower limbs (sensorimotor stimulation, passive, active-assisted and active mobilizations, exercises for muscle strength recovery, stretching, functional and task-oriented training), proprioceptive exercises, postural passages and transfers, sitting and standing training, motor coordination and balance training, walking training and activities of daily living recovery training. Moreover, all patients performed a robotic treatment of the upper limb session 5 times a week, lasting 45 min, using a set of robotic devices. Robotic treatment of the upper limb was based on the use of 4 robotic devices (Motore—Humanware srl, Pisa, Italy, and Amadeo, Diego and Pablo—Tyromotion GmBH, Graz, Austria), and it was focused on sensory stimulation, passive, active or assisted-active exercises, and task-oriented exercises of the upper limbs.

Patients were evaluated at baseline (T0) and re-evaluated after 6 weeks of rehabilitation treatment (T1) by means of the modified Barthel Index (BI), an ordinal scale used to measure performance in activities of daily living (ADL), ranging from 0 to 100, with lower scores indicating increased disability [42].

### 4.3. DNA Extraction

Genomic DNA was extracted from the peripheral blood (6 mL) of all patients with stroke at T0 and T1 time points using a Quick-DNA Midiprep Plus Kit (Zymo Research, Irvine, CA, USA) according to the manufacturer’s instructions. Genomic DNA was quantified by the Qubit 2.0 Fluorometer (ThermoFisher Scientific, Waltham, MA, USA) according to the manufacturer’s instructions.

### 4.4. BDNF rs6265 Polymorphism Genotyping

The *BDNF* rs6265 genotyping was performed using a polymerase chain reaction (PCR) combined with restriction enzyme digestion. An 84 base pairs (bp) fragment was amplified from 20 ng of genomic DNA (reaction volume of 20 μL), using a MyTaq DNA polymerase kit (Bioline, Taunton, MA, USA), and 10 mM of each primer (forward BD-F and reverse BD-R) (Santoro et al., 2016). The amplification conditions were as follows: 2 min at 95 °C, initial denaturation; 15 s at 95 °C, 30 s at 60 °C and 30 s at 72 °C for 35 cycles; 7 min at 72 °C, final extension. The PCR products, digested with 3 units of HpyCH4IV (cleavage sequence 5′-ACGT-3′) (New England Biolabs, Beverly, MA, USA) restriction enzyme, were resolved by electrophoresis on 2% agarose gels stained with Gel Red (Biotium, Fremont, CA, USA).

### 4.5. Bisulphite Conversion

In total, 2 µg of genomic DNA was bisulphite converted using an EZ DNA Methylation-Gold Kit (Zymo Research, Irvine, CA, USA) according to the manufacturer’s instructions. After bisulphite treatment, the genomic DNA was quantified by the Qubit 2.0 Fluorometer (ThermoFischer Scientific, Waltham, MA, USA) according to the manufacturer’s instructions.

### 4.6. Methylation Analysis of BDNF rs6265polymorphism

The methylation analysis was performed on the CpG site that can be created or abolished by *BDNF* rs6265 SNP using quantitative real-time PCR (qRT-PCR). qPCR was carried out using TaqMan probes previously described (Nociti et al., 2018).

Briefly, 20 ng of bisulfite-treated DNA was amplified with 10 μL of TaqMan Universal PCR Master Mix II, No AmpErase UNG (uracil-N-glycosylase) (ThermoFisher Scientific, Waltham, MA, USA), 0.4 μM each of the primers BD-1F and BD-1R and 0.2 μM each of the fluorescently labeled probes (FAM for methylated signal and VIC for unmethylated signal) [43].

qRT-PCR was performed on a StepOnePlus thermocycler (ThermoFisher Scientific, Waltham, MA, USA) and the amplification conditions were initial denaturation at 95 °C for 10 min followed by 40 cycles of denaturation at 95 °C for 15 s, and annealing and extension at 60 °C for 1 min.

The percentage of methylation was calculated considering the threshold cycles (Ct) for each dye. The methylated signal was provided by FAM channel (Ct_CG_) while the unmethylated signal was provided by VIC channel (Ct_TG_). Methylation percentage = 100/[1 + 2(Ct_CG_ − Ct_TG_)]% [44,45].

### 4.7. Statistics

The demographic and clinical characteristics of the enrolled sample are described as means and standard deviations, or percentage, as appropriate. The mean values of the methylation percentages in patients with different *BDNF* rs6265 genotype (GG, GA, and AA) were compared by using a one-way ANOVA, followed by post-hoc comparisons with Bonferroni correction.

Since the group of patients was very small, we conducted analysis considering patients carrying (A+) or not carrying (A−) the A allele.

In order to investigate the rehabilitation’s effects on the methylation percentages, we compared the values obtained at T0 with those at T1, for patients A+ and A−, separately, by using paired *t*-tests. Moreover, to analyze the relationship between the *BDNF* rs6265 polymorphism and the outcome of the rehabilitation intervention, we dichotomized the primary outcome, i.e., the modified Barthel Index (BI) after the rehabilitation intervention, into favorable and unfavorable. The BI cut-off scores were defined as BI ≥ 75 for a favorable outcome and as BI < 75 for an unfavorable outcome [46].

To analyze the relationship between methylation percentages and recovery, methylation percentages at T0 and T1, as well as the change (T1-T0), were compared between patients with favorable and unfavorable outcomes, by means of unpaired *t*-tests.

Finally, to identify possible predictors of recovery, we first performed a univariate analysis, aimed at evaluating the relationship (a) between the *BDNF* polymorphism (A+ or A−) and the rehabilitation outcome (favorable and unfavorable) through a chi-squared test, and (b) the methylation percentage at baseline and the rehabilitation outcome (favorable and unfavorable) through an unpaired *t*-test. Then, we performed a logistic regression, with the rehabilitation outcome as the dependent variable, the genetic/epigenetic variables significantly associated with the rehabilitation outcome as independent variables, and the age and the time since the stroke onset as possible confounders. For each statistical analysis, a *p*-value lower than 0.05 was deemed significant. Statistical analysis was performed using SPSS version 25 (IBM Corp., Armonk, NY, USA), while figures were made using GraphPad Prism version 8 (GraphPad Software, San Diego, CA, USA).

## Figures and Tables

**Table 1:** Demographic and clinical characteristics of the sample (*n* = 49).

Variable	Mean (SD), or Count (%)
Age	68.4 (14.3)
Sex	26 men (53.1%) 23 women (46.9%)
Time since stroke (days)	90.2 (31.2)
Type of stroke	36 ischemic (73.5%) 13 hemorrhagic (26.5%)
Hemiparesis side	20 right (40.8%) 29 left (59.2%)
Spatial Neglect	10 (26.3%)
Language impairment	9 (23.7%)
Modified Barthel Index	39.1 (17.2)

**Table 2:** Logistic regression model.

	B	SE	Wald	gl	P	OR	95% CI OR
Polymorphism *	1.72	0.87	3.93	1	0.047	5.59	1.02‒30.64
Time Since Stroke	−0.01	0.01	0.66	1	0.416	0.99	0.97‒1.01
Age	−0.03	0.02	1.31	1	0.252	0.97	0.93‒1.02
Constant	0.53	2.02	0.07	1	0.791	1.71	

* Ref = A+ allele.

**Table 3:** Methylation analysis of *BDNF* rs6265 SNP: comparison of methylation percentages in patients with or without a favorable outcome, together with the results of the statistical analysis (Welch’s unpaired *t*-test)**.**

Percentage of Methylation	Unfavorable Outcome		Favorable Outcome		*p*-Value
	Mean	SD	Mean	SD	
At Baseline	41.0	5.5	42.5	5.4	0.372
After the treatment	41.8	6.1	45.1	3.7	0.088
Changes from baseline	0.7	2.3	1.9	2.4	0.219
